# 2-Amino-3-nitro­pyridinium perchlorate

**DOI:** 10.1107/S1600536810000425

**Published:** 2010-01-09

**Authors:** Samah Toumi Akriche, Mohamed Rzaigui, Noura Al-Hokbany, Refaat Mohamed Mahfouz

**Affiliations:** a Laboratoire de Chimie des Matériaux, Faculté des Sciences de Bizerte, 7021 Zarzouna Bizerte, Tunisia; b Chemistry Department, Faculty of Science, King Saud University, PO Box 2455, Riyadh 11451, Saudi Arabia

## Abstract

The title compound, C_5_H_6_N_3_O_2_^+^·ClO_4_^−^, is comprised of discrete perchlorate anions and 2-amino-3-nitro­pyridinium cations. The anion has a typical tetra­hedral geometry while the cation presents a nearly planar [maximum deviation = 0.007 (8) Å] pyridinium ring. Undulating [C_5_H_6_N_3_O_2_^+^]_*n*_ chains extending along the *c*-axis direction are linked *via* N—H⋯O hydrogen bonds. The cations are further connected to the anions by N—H⋯O hydrogen bonds and weak C—H⋯O inter­actions, leading to the formation of a three-dimensional network.

## Related literature

For related structures, see: Akriche & Rzaigui (2000 ▶, 2009*a* ▶,*b* ▶,*c* ▶); Nicoud *et al.* (1997 ▶). For details of hydrogen bonding, see: Steiner & Saenger (1994 ▶). For bond lengths in related structures, see: Aakeröy *et al.* (1998 ▶); Messai *et al.* (2009 ▶).
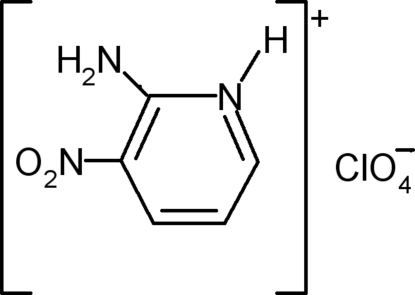


## Experimental

### 

#### Crystal data

C_5_H_6_N_3_O_2_^+^·ClO_4_^−^
*M* *_r_* = 239.58
Monoclinic, 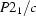

*a* = 5.888 (2) Å
*b* = 18.342 (6) Å
*c* = 9.170 (4) Å
β = 116.61 (3)°
*V* = 885.3 (6) Å^3^
*Z* = 4
Mo *K*α radiation
μ = 0.45 mm^−1^
*T* = 293 K
0.29 × 0.25 × 0.21 mm

#### Data collection

Enraf–Nonius TurboCAD-4 diffractometer
Absorption correction: multi-scan (Blessing, 1995 ▶) *T*_min_ = 0.725, *T*_max_ = 0.912
3574 measured reflections
2130 independent reflections
1109 reflections with *I* > 2σ(*I*)
*R*_int_ = 0.046
2 standard reflections every 120 min  intensity decay: 1%

#### Refinement

*R*[*F* ^2^ > 2σ(*F* ^2^)] = 0.062
*wR*(*F* ^2^) = 0.189
*S* = 1.00
2130 reflections
136 parameters
66 restraints
H-atom parameters constrained
Δρ_max_ = 0.52 e Å^−3^
Δρ_min_ = −0.30 e Å^−3^

### 

Data collection: *CAD-4 EXPRESS* (Enraf–Nonius, 1994 ▶); cell refinement: *CAD-4 EXPRESS*; data reduction: *XCAD4* (Harms & Wocadlo, 1995 ▶); program(s) used to solve structure: *SHELXS86* (Sheldrick, 2008 ▶); program(s) used to refine structure: *SHELXL97* (Sheldrick, 2008 ▶); molecular graphics: *ORTEP-3 for Windows* (Farrugia, 1997 ▶) and *DIAMOND* (Brandenburg & Putz, 2005 ▶); software used to prepare material for publication: *WinGX* (Farrugia, 1999 ▶).

## Supplementary Material

Crystal structure: contains datablocks I, global. DOI: 10.1107/S1600536810000425/pv2249sup1.cif

Structure factors: contains datablocks I. DOI: 10.1107/S1600536810000425/pv2249Isup2.hkl

Additional supplementary materials:  crystallographic information; 3D view; checkCIF report

## Figures and Tables

**Table 1:** Hydrogen-bond geometry (Å, °)

*D*—H⋯*A*	*D*—H	H⋯*A*	*D*⋯*A*	*D*—H⋯*A*
N1—H1⋯O1	0.86	2.28	2.927 (5)	133
N1—H1⋯O1^i^	0.86	2.44	2.969 (5)	121
N2—H2*A*⋯O2^ii^	0.86	2.03	2.886 (5)	173
N2—H2*B*⋯O5	0.86	2.04	2.633 (5)	126
N2—H2*B*⋯O6^iii^	0.86	2.32	2.917 (5)	126
C5—H5⋯O3^iv^	0.93	2.57	3.270 (5)	133

Symmetry codes: (i) 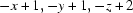; (ii) 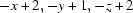; (iii) 
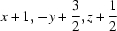
; (iv) 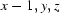.

**Fig. 1.**
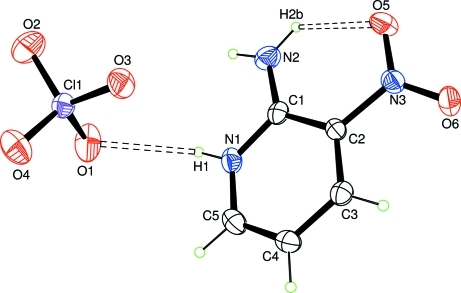
An ORTEP view of (I) with the atom-labelling scheme. Displacement ellipsoids are drawn at the 30% probability level. H atoms are represented by spheres of arbitrary radii. Hydrogen bonds are represented as dashed lines.

**Fig. 2.**
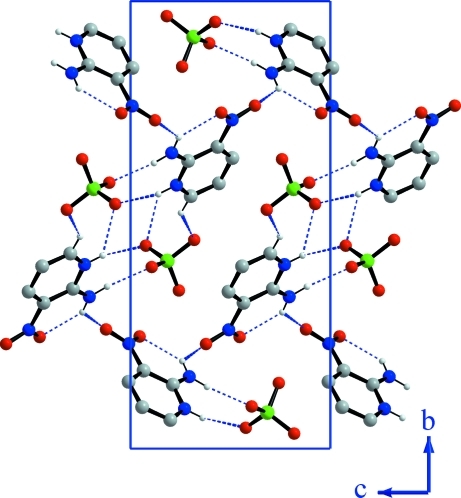
Projection of (I) down the a axis. The H-atoms not involved in H-bonding are omitted.

## supplementary crystallographic information

### Comment

Salts of 2-amino-3-nitropyridine attracted more attention as non linear optical (NLO) materials after discovering the promising properties of 2-amino-3-nitropyridinium chloride (Nicoud *et al.*,1997). With the purpose of obtaining non-centrosymmetric crystals of 2-amino-3-nitropyridine salts, its interaction with various acids has been studied and we have elaborated a serie of new materials with this organic molecule (Akriche & Rzaigui, 2000; Akriche & Rzaigui, 2009a; 2009b; 2009c). In this paper, we describe the crystal structure of the title compound (I).

The asymmetric unit of (I) is composed of a perchlorate anion and a 2-amino-3-nitropyridinium (2 A3NP) cation (Fig. 1). The anions are surrounded by two cations *via* hydrogen bonds which play an important role in stabilizing the crystal structure (Fig. 2). In the crystal structure, one can distinguish the ondulated chains of the cations extending along the *c* axis. The adjacent cations are joined by the N2—H2B···O6 (Table 1) hydrogen bond with N···O distance of 2.917 (5) Å. The cations are also connected to the chlorate anions by hydrogen bonds, of the type N—H···O with N···O distances in the range 2.886 (5) - 2.969 (5) Å, and weak C5—H5···O3 interaction with C5···O3 separation 3.270 (5) Å (Fig. 2, Table 1). The C—H···O bonds have already been evidenced by several authors in molecular crystals (Steiner *et al.*, 1994).

The anion displays a typical tetrahedral geometry around Cl atom and the Cl···O distances compare well with previously reported values (Messai *et al.*, 2009). The Cl—O bond distances and O—Cl—O bond angles (Table: Geometric parameters) confirm a tetrahedral conformation, similar to other perchlorates quoted above.

The pyridinium ring of the cation is nearly planar, with maximum deviation from planarity being 0.007 (8) Å for C1 atom. The diedral angle between the planes of the NO_2_ group and the pyridinium ring is 9.7 (2) ° indicating a deviation of the NO_2_ group from being co-planar with the ring since its oxygen atoms are involved in various types of inter- and intramolecular hydrogen bonds. Moreover, the C—NH_2_ (1.313 (5) Å) and C—NO_2_ (1.448 (5) Å) distances in the 2 A3NP cation are respectively shortened and lengthened with respect to the C—NH_2_ (1.337 (4) Å) and C—NO_2_ (1.429 (4) Å) observed in the crystal structure of 2-amino-3-nitropyridine  (Aakeröy *et al.*, 1998). All the 2-amino-3-nitropyridinium cations hosted in various organic or inorganic matrices show the same changes in C—NH_2_ and C—NO_2_ distances, revealing a weak increase of π bond character in C—NH_2_ and a decrease in C—NO_2_. The bond lengths of cation in (I) are normal and comparable with the corresponding values observed in the related structure (Akriche & Rzaigui, 2000; Akriche & Rzaigui, 2009a; 2009b, 2009c).

### Experimental

2-Amino-3-nitropyridine (4 mmol, 354 mg) was dissolved in a solution of perchloride acid (4 mmol in 20 ml water). The mixture was stirred for about 30 min at 333 K and evaporated in the air giving colorless block crystals of the title compound suitable for X-ray analysis.

### Refinement

H atoms were treated as riding, with C—H = 0.93 A ° and N—H = 0.86 A ° and with *U*_iso_(H) = 1.2Ueq(C or N). The atoms of the chlorate ion were refined using isotropic U_ij_ restraints.

### Crystal data

**Table:**

C_5_H_6_N_3_O_2_^+^·ClO_4_^−^	*F*(000) = 488
*M**_r_* = 239.58	*D*_x_ = 1.797 Mg m^−^^3^
Monoclinic, *P*2_1_/*c*	Mo *K*α radiation, λ = 0.71073 Å
Hall symbol: -P 2ybc	Cell parameters from 25 reflections
*a* = 5.888 (2) Å	θ = 9–11°
*b* = 18.342 (6) Å	µ = 0.45 mm^−^^1^
*c* = 9.170 (4) Å	*T* = 293 K
β = 116.61 (3)°	Prism, colorless
*V* = 885.3 (6) Å^3^	0.29 × 0.25 × 0.21 mm
*Z* = 4	

### Data collection

**Table:**

Enraf–Nonius TurboCAD-4 diffractometer	1109 reflections with *I* > 2σ(*I*)
Radiation source: fine-focus sealed tube	*R*_int_ = 0.046
graphite	θ_max_ = 28.0°, θ_min_ = 2.2°
Non–profiled ω scans	*h* = −7→7
Absorption correction: multi-scan (Blessing, 1995)	*k* = −24→0
*T*_min_ = 0.725, *T*_max_ = 1.101	*l* = −11→12
3574 measured reflections	2 standard reflections every 120 min
2130 independent reflections	intensity decay: 1%

### Refinement

**Table:**

Refinement on *F*^2^	Primary atom site location: structure-invariant direct methods
Least-squares matrix: full	Secondary atom site location: difference Fourier map
*R*[*F*^2^ > 2σ(*F*^2^)] = 0.062	Hydrogen site location: inferred from neighbouring sites
*wR*(*F*^2^) = 0.189	H-atom parameters constrained
*S* = 1.00	*w* = 1/[σ^2^(*F*_o_^2^) + (0.096*P*)^2^] where *P* = (*F*_o_^2^ + 2*F*_c_^2^)/3
2130 reflections	(Δ/σ)_max_ < 0.001
136 parameters	Δρ_max_ = 0.52 e Å^−^^3^
66 restraints	Δρ_min_ = −0.30 e Å^−^^3^

### Special details

**Table:**

Geometry. All e.s.d.'s (except the e.s.d. in the dihedral angle between two l.s. planes) are estimated using the full covariance matrix. The cell e.s.d.'s are taken into account individually in the estimation of e.s.d.'s in distances, angles and torsion angles; correlations between e.s.d.'s in cell parameters are only used when they are defined by crystal symmetry. An approximate (isotropic) treatment of cell e.s.d.'s is used for estimating e.s.d.'s involving l.s. planes.
Refinement. Refinement of *F*^2^ against ALL reflections. The weighted *R*-factor *wR* and goodness of fit *S* are based on *F*^2^, conventional *R*-factors *R* are based on *F*, with *F* set to zero for negative *F*^2^. The threshold expression of *F*^2^ > σ(*F*^2^) is used only for calculating *R*-factors(gt) *etc*. and is not relevant to the choice of reflections for refinement. *R*-factors based on *F*^2^ are statistically about twice as large as those based on *F*, and *R*- factors based on ALL data will be even larger.

### Fractional atomic coordinates and isotropic or equivalent isotropic displacement parameters (Å^2^)

**Table:**

	*x*	*y*	*z*	*U*_iso_*/*U*_eq_	
O1	0.6409 (9)	0.45343 (19)	0.9234 (4)	0.0967 (12)	
O2	0.9810 (6)	0.4058 (2)	0.8940 (5)	0.1130 (15)	
O3	0.6657 (6)	0.46634 (17)	0.6814 (4)	0.0750 (9)	
O4	0.5876 (7)	0.35339 (19)	0.7581 (5)	0.0970 (12)	
N1	0.3798 (6)	0.58877 (17)	0.7702 (4)	0.0563 (8)	
H1	0.4836	0.5686	0.8602	0.068*	
N2	0.7098 (6)	0.6608 (2)	0.7939 (4)	0.0663 (10)	
H2A	0.8051	0.6381	0.8826	0.080*	
H2B	0.7720	0.6953	0.7592	0.080*	
N3	0.3603 (7)	0.73452 (18)	0.4915 (4)	0.0562 (9)	
C1	0.4694 (7)	0.6428 (2)	0.7117 (4)	0.0453 (9)	
C2	0.2906 (7)	0.67427 (18)	0.5653 (4)	0.0419 (8)	
C3	0.0457 (7)	0.6493 (2)	0.4903 (5)	0.0498 (9)	
H3	−0.0702	0.6705	0.3932	0.060*	
C4	−0.0302 (7)	0.5927 (2)	0.5582 (5)	0.0544 (10)	
H4	−0.1957	0.5750	0.5077	0.065*	
C5	0.1418 (9)	0.5641 (2)	0.6989 (5)	0.0591 (11)	
H5	0.0944	0.5265	0.7475	0.071*	
O5	0.5840 (6)	0.75060 (17)	0.5457 (4)	0.0767 (9)	
O6	0.1928 (6)	0.76632 (17)	0.3776 (4)	0.0757 (9)	
Cl1	0.71827 (18)	0.41956 (5)	0.81544 (11)	0.0530 (3)	

### Atomic displacement parameters (Å^2^)

**Table:**

	*U*^11^	*U*^22^	*U*^33^	*U*^12^	*U*^13^	*U*^23^
O1	0.161 (4)	0.083 (2)	0.076 (2)	0.040 (2)	0.079 (2)	0.0169 (19)
O2	0.057 (2)	0.133 (4)	0.119 (3)	0.022 (2)	0.013 (2)	0.029 (3)
O3	0.090 (2)	0.078 (2)	0.0663 (19)	0.0100 (17)	0.0439 (17)	0.0197 (16)
O4	0.116 (3)	0.066 (2)	0.111 (3)	−0.024 (2)	0.052 (2)	−0.009 (2)
N1	0.068 (2)	0.0509 (19)	0.0523 (19)	0.0097 (18)	0.0286 (17)	0.0124 (16)
N2	0.048 (2)	0.077 (3)	0.065 (2)	0.0020 (18)	0.0174 (17)	0.003 (2)
N3	0.064 (2)	0.0459 (19)	0.070 (2)	0.0006 (18)	0.040 (2)	0.0018 (17)
C1	0.052 (2)	0.044 (2)	0.044 (2)	0.0093 (17)	0.0256 (18)	−0.0033 (16)
C2	0.054 (2)	0.0345 (17)	0.048 (2)	0.0019 (16)	0.0320 (18)	−0.0017 (15)
C3	0.051 (2)	0.052 (2)	0.046 (2)	0.0064 (18)	0.0219 (18)	0.0028 (18)
C4	0.053 (2)	0.055 (2)	0.062 (3)	−0.0066 (19)	0.032 (2)	−0.004 (2)
C5	0.078 (3)	0.049 (2)	0.065 (3)	−0.006 (2)	0.045 (2)	0.000 (2)
O5	0.073 (2)	0.067 (2)	0.103 (2)	−0.0138 (18)	0.0520 (19)	0.0072 (18)
O6	0.085 (2)	0.064 (2)	0.086 (2)	0.0191 (18)	0.0458 (19)	0.0309 (18)
Cl1	0.0554 (6)	0.0519 (6)	0.0498 (6)	0.0047 (5)	0.0219 (4)	0.0041 (5)

### Geometric parameters (Å, °)

**Table:**

O1—Cl1	1.406 (3)	N3—O6	1.216 (4)
O2—Cl1	1.406 (3)	N3—O5	1.218 (4)
O3—Cl1	1.415 (3)	N3—C2	1.448 (5)
O4—Cl1	1.407 (3)	C1—C2	1.406 (5)
N1—C5	1.332 (5)	C2—C3	1.369 (5)
N1—C1	1.343 (5)	C3—C4	1.384 (5)
N1—H1	0.8600	C3—H3	0.9300
N2—C1	1.313 (5)	C4—C5	1.339 (6)
N2—H2A	0.8600	C4—H4	0.9300
N2—H2B	0.8600	C5—H5	0.9300
			
C5—N1—C1	124.8 (3)	C2—C3—C4	120.3 (4)
C5—N1—H1	117.6	C2—C3—H3	119.8
C1—N1—H1	117.6	C4—C3—H3	119.8
C1—N2—H2A	120.0	C5—C4—C3	118.0 (4)
C1—N2—H2B	120.0	C5—C4—H4	121.0
H2A—N2—H2B	120.0	C3—C4—H4	121.0
O6—N3—O5	123.1 (3)	N1—C5—C4	121.0 (4)
O6—N3—C2	118.5 (3)	N1—C5—H5	119.5
O5—N3—C2	118.4 (3)	C4—C5—H5	119.5
N2—C1—N1	118.1 (4)	O2—Cl1—O1	110.3 (3)
N2—C1—C2	126.8 (4)	O2—Cl1—O4	109.2 (3)
N1—C1—C2	115.1 (3)	O1—Cl1—O4	110.4 (2)
C3—C2—C1	120.8 (3)	O2—Cl1—O3	108.4 (2)
C3—C2—N3	118.4 (3)	O1—Cl1—O3	109.26 (19)
C1—C2—N3	120.8 (4)	O4—Cl1—O3	109.2 (2)
			
C5—N1—C1—N2	−179.1 (4)	O6—N3—C2—C1	−170.3 (3)
C5—N1—C1—C2	0.9 (5)	O5—N3—C2—C1	10.0 (5)
N2—C1—C2—C3	178.9 (3)	C1—C2—C3—C4	0.3 (5)
N1—C1—C2—C3	−1.1 (5)	N3—C2—C3—C4	−179.0 (3)
N2—C1—C2—N3	−1.8 (6)	C2—C3—C4—C5	0.7 (6)
N1—C1—C2—N3	178.3 (3)	C1—N1—C5—C4	0.1 (6)
O6—N3—C2—C3	9.0 (5)	C3—C4—C5—N1	−0.9 (6)
O5—N3—C2—C3	−170.7 (3)		

### Hydrogen-bond geometry (Å, °)

**Table:**

*D*—H···*A*	*D*—H	H···*A*	*D*···*A*	*D*—H···*A*
N1—H1···O1	0.86	2.28	2.927 (5)	133
N1—H1···O1^i^	0.86	2.44	2.969 (5)	121
N2—H2A···O2^ii^	0.86	2.03	2.886 (5)	173
N2—H2B···O5	0.86	2.04	2.633 (5)	126
N2—H2B···O6^iii^	0.86	2.32	2.917 (5)	126
C5—H5···O3^iv^	0.93	2.57	3.270 (5)	133

Symmetry codes: (i) −*x*+1, −*y*+1, −*z*+2; (ii) −*x*+2, −*y*+1, −*z*+2; (iii) *x*+1, −*y*+3/2, *z*+1/2; (iv) *x*−1, *y*, *z*.
